# Pleural fluid cell-free DNA integrity index to identify cytologically negative malignant pleural effusions including mesotheliomas

**DOI:** 10.1186/1471-2407-12-428

**Published:** 2012-09-25

**Authors:** Krishna B Sriram, Vandana Relan, Belinda E Clarke, Edwina E Duhig, Morgan N Windsor, Kevin S Matar, Rishendran Naidoo, Linda Passmore, Elizabeth McCaul, Deborah Courtney, Ian A Yang, Rayleen V Bowman, Kwun M Fong

**Affiliations:** 1 UQ Thoracic Research Centre, School of Medicine, The University of Queensland, Queensland, Australia; 2 Department of Thoracic Medicine, The Prince Charles Hospital, Queensland, Australia; 3 Department of Anatomical Pathology, The Prince Charles Hospital, Queensland, Australia; 4 Department of Thoracic Surgery, The Prince Charles Hospital, QLD, Australia

**Keywords:** Malignant pleural effusions, Mesothelioma, Lung cancer, DNA integrity index, Mesothelin

## Abstract

**Background:**

The diagnosis of malignant pleural effusions (MPE) is often clinically challenging, especially if the cytology is negative for malignancy. DNA integrity index has been reported to be a marker of malignancy. The aim of this study was to evaluate the utility of pleural fluid DNA integrity index in the diagnosis of MPE.

**Methods:**

We studied 75 pleural fluid and matched serum samples from consecutive subjects. Pleural fluid and serum ALU DNA repeats [115bp, 247bp and 247bp/115bp ratio (DNA integrity index)] were assessed by real-time quantitative PCR. Pleural fluid and serum mesothelin levels were quantified using ELISA.

**Results:**

Based on clinico-pathological evaluation, 52 subjects had MPE (including 16 mesotheliomas) and 23 had benign effusions. Pleural fluid DNA integrity index was higher in MPE compared with benign effusions (1.2 vs. 0.8; *p*<0.001). Cytology had a sensitivity of 55% in diagnosing MPE. If cytology and pleural fluid DNA integrity index were considered together, they exhibited 81% sensitivity and 87% specificity in distinguishing benign and malignant effusions. In cytology-negative pleural effusions (35 MPE and 28 benign effusions), elevated pleural fluid DNA integrity index had an 81% positive predictive value in detecting MPEs. In the detection of mesothelioma, at a specificity of 90%, pleural fluid DNA integrity index had similar sensitivity to pleural fluid and serum mesothelin (75% each respectively).

**Conclusion:**

Pleural fluid DNA integrity index is a promising diagnostic biomarker for identification of MPEs, including mesothelioma. This biomarker may be particularly useful in cases of MPE where pleural aspirate cytology is negative, and could guide the decision to undertake more invasive definitive testing. A prospective validation study is being undertaken to validate our findings and test the clinical utility of this biomarker for altering clinical practice.

## Background

Pleural effusions can be caused by a diverse array of pleural, pulmonary or extrapulmonary diseases [1,2]. An important diagnosis to be established or excluded is malignancy, particularly in unilateral pleural effusions. More than 75% of malignant pleural effusions (MPEs) worldwide are due to metastases from tumours originating in the lung and breast or lymphoma. Less commonly, MPEs are due to mesothelioma, which usually arises after a long latent period after exposure to asbestos fibres. The median survival of patients with MPEs is only 4-10 months after clinical diagnosis. Consequently, expert guidelines recommend that when investigating pleural effusions, a minimum number of tests be performed while aiming to achieve a swift diagnosis [3]. Almost all patients with unilateral pleural effusions undergo pleural fluid aspiration and cytology. While cytology remains the analytical method of choice for the detection of tumour cells in pleural fluid [4], its sensitivity varies between 30% for mesothelioma to up to 60% for adenocarcinoma [5,6].

Recently, soluble mesothelin-related peptide (or mesothelin), has been approved by the U.S. Food and Drug Administration for the diagnosis and monitoring of mesothelioma. Mesothelin is a glycoprotein overexpressed by mesothelioma and measurements in pleural fluid and serum are significantly higher compared to non-mesothelioma MPEs and benign effusions [7,8]. While pleural fluid and serum mesothelin are diagnostic biomarkers for mesothelioma, a similar biomarker for non-mesothelioma MPEs may have considerable clinical utility.

Cell-free DNA (cfDNA) is a macromolecule that can be readily detected in biological fluids and is believed to be released from either apoptotic or necrotic cells [9]. Necrosis, which occurs in malignant tumours typically generates a spectrum of DNA fragments with varying strand lengths due to random and incomplete digestion of genomic DNA by deoxyribonucleases [10]. In contrast, cell death in normal nucleated blood cells occurs predominantly via apoptosis resulting in the production of uniform small DNA fragments (<200bp) [11]. The DNA integrity index, measured as the ratio of longer to shorter DNA fragments, has been shown to be higher in the plasma and serum of patients with solid organ malignancies compared to normal individuals [12]. Hence, the measurement of DNA integrity index in biological fluids has promise as a minimally invasive diagnostic biomarker for malignancy. To the best of our knowledge, the utility of DNA integrity in diagnosing MPE and specifically mesothelioma has not been compared to pleural fluid cytology and mesothelin.

In this study, we measured DNA fragments by real-time quantitative polymerase chain reaction (PCR) on 75 matched pleural fluid and serum samples. We chose to measure ALU sequences since they are the most abundant repetitive sequences accounting for more than 10% of the genome. Our primary objective was to determine if pleural fluid and/or serum DNA integrity could serve as a diagnostic biomarker for MPEs, particularly where cytological examination is negative and clinical suspicion remains. A secondary objective of the study was to compare DNA integrity index to mesothelin levels measured by ELISA assay in pleural fluid and serum for diagnosis of mesothelioma.

## Methods

### Subjects and sample collection

We recruited 75 consecutive subjects with undiagnosed pleural effusions referred to The Prince Charles Hospital (Brisbane, Australia) between February 2010 and September 2011. All study subjects provided written informed consent. The study was approved by the Human Research Ethics Committees at The Prince Charles Hospital (TPCH) and The University of Queensland. Pleural fluid was collected during simple thoracocentesis, performed during the diagnostic work-up of patients with pleural effusions or prior to tube thoracostomy, or a surgical thoracoscopic procedure. In all but one subject, pleural fluid obtained at the time of the first thoracocentesis was used for analysis. The pleural fluid was transferred within 24 hours to the laboratory in polystyrene containers without anticoagulant (SARSTEDT, Nümbrecht, Germany) at room temperature. The pleural fluid samples were centrifuged for 7 minutes at 600g and supernatants stored in 1ml aliquots at -80°C for DNA extraction and ELISA experiments. Matched blood samples were also collected within 24 hours of obtaining pleural fluid. Serum was separated by centrifuging the blood samples for 10 minutes at 1700g and stored at -20°C for DNA extraction and ELISA experiments. Two mL of pleural fluid and serum were used for DNA extraction using Nucleospin kits (Machery-Nagel, DÜREN, Germany) following the manufacturer’s instructions. DNA was eluted in 100μL TE buffer and stored at -80°C until use. Mesothelin ELISA assays were performed on pleural fluid supernatant and serum aliquots stored at -80°C and -20°C respectively, then allowed to thaw to room temperature.

### Measurement of DNA fragments

Quantification of DNA fragments was performed by quantitative real-time PCR (qPCR) of ALU 115bp and 247bp repeats as previously published [13]. The sequences of the ALU 115bp primers were as follows: forward: 5^′^-CCTGAGGTCAGGAGTTCGAG-3^′^ and reverse: 5^′^-CCCGAGTAGCTGGGATTACA-3^′^; ALU 247bp primers were forward: 5^′^-GTGGCTCACGCCTGTAATC-3^′^ and reverse: 5^′^-CAGGCTGGAGTGCAGTGG-3^′^.

**Figure 1:**
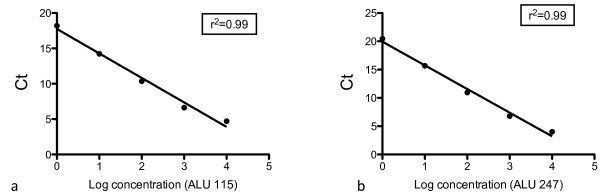
**Standard curves of ALU 115bp and 247bp amplicons.** DNA from female genomic DNA was serially diluted (from 10ng to 0.1ng) and used to construct the standard curves from which the quantity of DNA of the patients was calculated.

**Figure 2:**
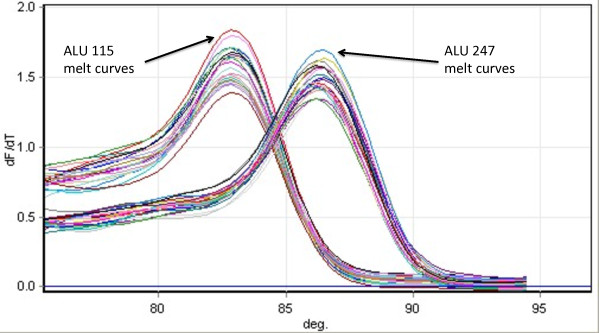
**Melting curves of ALU 115bp and ALU 247bp amplifications signals from 20 patients.** Following amplification, melting curves were obtained by calculating the derivatives dF/dT vs. temperature. ALU 115bp primers produce a peak of approximately 83°C and ALU 247bp at approximately 86°C.

The ALU 115 primer set amplifies both short 115bp product while the ALU 247 primer set amplified a longer 247bp fragments. The amplicon sizes were confirmed on gel electrophoresis. Quantification of DNA in each sample was determined by a standard curve with serial dilutions (10ng-0.01pg) of commercially available human female genomic DNA (Promega, Sydney, Australia) (Figure 1). Standard curves were determined for both ALU 115 and 247 primer sets respectively and the curves were obtained by putting the concentration log of the standard in x-axis and the values of the threshold cycles (Ct) in the y-axis. The quantity of DNA (ng/ml) present in the sample was extrapolated from the standard curve according to the Ct value. The DNA integrity index, represented by the ratio of the longer to shorter (ALU 247bp/ALU 115bp) fragments, was calculated for each individual sample (pleural effusion and serum) by dividing the ALU247 DNA quantity (ng/mL) by the ALU 115 DNA quantity (ng/mL). A negative control (water template) was performed in each plate. 10ng of commercially available human female genomic DNA (Promega, Sydney, Australia) was used as a positive control in each plate run. The reaction mixture consisted of 1μl of DNA template, 20μmol/L of the forward and reverse primers (ALU115 or ALU247), 5μl of QuantiFast mastermix (Qiagen, Sydney, Australia) in a total reaction volume of 10μl. Real-time PCR amplification was performed with hold at 95°C for 10 minutes, followed by 35 cycles of 95°C for 30s and 64°C for 30s, followed by melt curve analysis using a RotorGene 6000 (Qiagen, Hilden, Germany). The PCR protocol was optimised to yield optimal results to suit the equipment, reagents and conditions of our laboratory. Laboratory personnel performing the qPCR assays were blinded to the specimen identity. Using the human female genomic DNA (positive control) we found that the median intra- and inter-assay coefficient of variation (CV) for 115bp were 4% (IQR, 3%-6%) and 4% (IQR, 3%-4%) respectively. Similarly, the median intra- and inter- assay CV of human female genomic DNA for the 247bp primers were 4% (IQR 3%-7%) and 6% (IQR, 5%-7%) respectively. Melt curve analysis demonstrated that the 115bp amplicon produced a peak at 83°C and the 247bp amplicon produced a peak at 86°C (Figure 2).

### Mesothelin assay

Pleural fluid and serum mesothelin concentrations were determined in duplicate using a double-determinant ELISA assay, the MESOMARK kit (Fujirebio Diagnostics, Malvern, PA, USA), according to the manufacturer’s instructions. Nanomolar (nM) mesothelin concentrations were determined from a standard curve performed on each plate. Samples with high (13.5nM, range 10.8-16.2nM) and low (4.5nM, range: 3.6-5.4nM) mesothelin concentrations provided in the MESOMARK kit were run on each plate in duplicate to check inter-assay reproducibility. The inter-assay and intra-assay CV of mesothelin concentration of the positive control samples were 3% (IQ 2%-4%) and 4% (2%-5%) respectively.

### Diagnoses of pleural effusions

A definitive diagnosis of the pleural effusion was obtained by interrogation of the subject’s hospital records and pathology database. In brief, Light’s criteria were used to differentiate transudates from exudates [14]. The effusion was categorized as MPE if the pleural fluid cytology was positive for malignancy. Subjects with negative pleural fluid cytology were also deemed to have MPEs if a tissue biopsy was positive for malignancy or if subjects had disseminated malignancy and there was no alternative explanation for the effusion. Benign effusions were considered to be due to: ‘inflammatory pleuritis’ in subjects with histologic evidence of pleural inflammation only; ‘parapneumonic’ in subjects with an exudative effusion and clinical evidence of pneumonia; ‘asbestos related effusion’ in subjects with asbestos exposure, pleural plaques and/or rounded atelectasis and no evidence of mesothelioma; ‘congestive cardiac failure’ in subjects with transudative effusions and clinical features of cardiac failure; ‘chronic liver disease’ in a subject with a transudate effusion and previously diagnosed cirrhosis; ‘pulmonary embolism’ in a subject with an exudative effusion and demonstration of pulmonary embolism on computed tomography pulmonary angiogram and ‘connective tissue disorder’ in a subject with an exudative effusion and previously diagnosed mixed connective tissue disorder.

### Statistical analysis

The Mann-Whitney *U* test was used to compare total and necrotic DNA, integrity index and mesothelin in pleural fluid and serum between subjects with MPE and those with benign effusions. Spearman’s rho coefficient was used to assess correlations between mesothelin values, total DNA, necrotic DNA and DNA integrity index in serum and pleural effusions. A receiver operating characteristic (ROC) curve was performed to determine the area under the curve, sensitivity and specificity of pleural fluid DNA integrity index and mesothelin in the pleural fluid and serum. The area under the ROC curve (AUC) was computed with 95% confidence intervals. All statistical tests were performed using SPSS Student Version 19 (SPSS Inc., Chicago, IL, USA) and results were considered significant at *p* <0.05 (two-tailed).

## Results

### Subject characteristics

Based on clinical and pathological review, 52 study subjects were deemed to have a MPE while 23 subjects had benign effusions (Table 1). The most common aetiologies of MPE were lung cancer (n=22), mesothelioma (n=16), breast cancer (n=5) and lymphoma (n=3), while the most common aetiologies for benign effusions were inflammatory pleuritis (n=8), asbestos related effusions (n=4), parapneumonic effusions (n=4) and congestive cardiac failure (n=4) (Table 2). The clinico-pathological characteristics of the study subjects are listed in Table 2. The median age of the MPE and benign effusions subjects was 68 years and 73 years respectively. There were no differences in the characteristics of patients between MPE and benign effusions. Male subjects comprised 60% of the MPE group compared to 74% in the benign effusion group. Pleural fluid cytology analysis identified malignant cells in twenty-nine patients (56%). Among the remaining 23 patients with MPEs, the diagnosis was established by pleural biopsy in 16 patients. For the remaining seven patients, the treating clinician determined the pleural effusion to be malignant in the context of disseminated malignancy. Five patients with transudate effusions were determined to be MPEs, of which 1 patient was cytology positive while the remaining four disseminated malignancy.

**Table 1:** Aetiology of pleural effusions

	**Number of Patients, n(%)**
**Malignant Pleural Effusions**	
Lung cancer	22 (42)
Mesothelioma	16 (31)
Breast carcinoma	5 (10)
Lymphoma	3 (6)
Renal cell carcinoma	1 (2)
Melanoma	1 (2)
Peripheral nerve sheath tumour	1 (2)
Solitary fibrous tumour	1 (2)
Prostate carcinoma	1 (2)
Gastric adenocarcinoma	1 (2)
**Benign Pleural Effusions**	
Inflammatory pleuritis	8 (35)
Asbestos related effusions	4 (17)
Parapneumonic	4 (17)
Congestive cardiac failure	4 (17)
Chronic liver disease	1 (4)
Pulmonary embolism	1 (4)
Connective tissue disorder	1 (4)

**Table 2:** Clinicopathological characteristics of study subjects

	**Malignant pleural effusion N = 52**	**Benign pleural effusion N = 23**	***p*-value**
Age, years			
Median	68	73	
Range	30 – 94	41 – 92	
< 60 years of age	12 (23)	4 (17)	0.762
> 60 years of age	40 (77)	19 (83)	
Gender, n (%)			
Male	31 (60)	17 (74)	0.301
Female	21 (40)	6 (26)	
Smoking status, n (%)			
Current or Former	33 (63)	17 (84)	0.435
Never	19 (37)	6 (26)	
Pleural fluid, median (IQR)			
Protein, g/L	43.5 (37.0 – 48.0)	40 (31.8 – 45.8)	
LDH, IU/L	390 (198.5 – 926.5)	222 (168 – 322)	
Glucose, mmol/L	6 (4 -7)	6 (4 – 8)	
Light’s criteria classification, n (%)			
Transudate	5 (10)	2 (9)	1.00
Exudate	47 (90)	21 (91)	
Pleural fluid cytology, n (%)			
Positive for malignancy	29 (56)	0 (0)	<0.001
Negative for malignancy	23 (44)	23 (100)	

### Quantification of cell-free DNA fragments

#### Pleural Fluid

**Figure 3:**
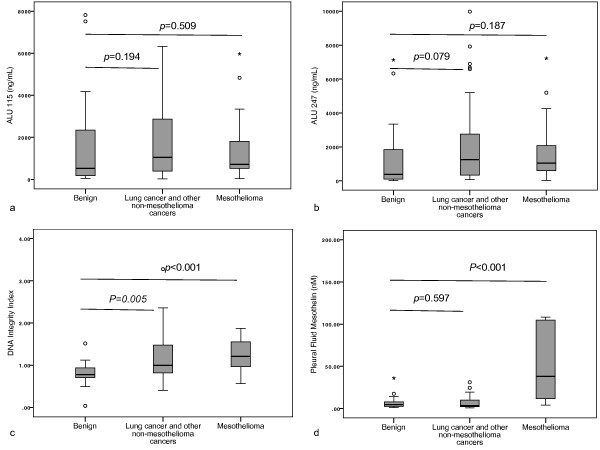
**Box plots of pleural effusion DNA fragments, integrity index and mesothelin concentrations in subjects with effusions due to benign aetiology, other cancers and mesothelioma.** DNA fragment values were determined by quantitative PCR for ALU (**a**) 115bp fragment and (**b**) a 247bp fragment. The DNA integrity index (**c**) was defined as the ratio of ALU 247/ALU 115 fragment levels. Mesothelin values (**d**) were determined by ELISA. Mann-Whitney *U* test to was used to assess for any differences in comparison to benign effusions and *p* values <0.05 were deemed significant.

Pleural fluid DNA fragment analysis for MPE and benign effusions are provided in Table 3. There was no difference between MPE and benign effusions in the pleural fluid ALU 115 (median, 963.9 ng/mL vs. 520.3 ng/mL, *p*=0.217) (Figure 3a), or ALU 247 (median, 1186.2 ng/mL vs. 371.8 ng/mL, *p*=0.066) (Figure 3b). However the DNA integrity index was higher in the MPEs compared to benign effusions (median, 1.1 vs. 0.8, *p*<0.001) (Figure 3c). Furthermore DNA integrity index was higher than benign effusions in all subgroups of MPE, i.e. mesothelioma (median, 1.2 vs. 0.8, *p*<0.001), lung cancer (median, 1.09 vs. 0.8, *p*=0.018) and other metastatic cancers (median, 1.3 vs. 0.8, *p*<0.001).

**Table 3:** Pleural fluid DNA fragments, integrity index and mesothelin in subjects with malignant and benign pleural effusions

		**All MPE(*n*= 52)**	**Malignant pleural mesothelioma only (n = 16)**	**Lung cancer (n=22)**	**Other cancers (n=14)**	**BPE(*n*= 23)**
ALU 115 (ng/ml)	*p*-value	0.217	0.509	0.147	0.526	
	Mean, Range	1841.6	1512.2	2065.0	1836.6	1616.7
		(21.8-6328.7)	(41.7 – 5964.2)	(21.8 – 6204.8)	(176.5 – 6328.7)	(48.5 – 7814.9)
	Median (IQR 25-75)	963.9	712.3	1366.5	723.5	520.3
		(411.1-2385.7)	(502.3 – 1992.9)	(488.4 – 2624.0)	(299.1 – 4492.1)	(128.6 – 2481.9)
ALU 247 (ng/ml)	*p*-value	0.066	0.187	0.061	0.231	
	Mean, Range	2065.7	1827.6	1947.8	2694.1	1389.4
		(23.5 – 9973.7)	(23.5 – 7220)	(71.5 – 6671.4)	(151.7 – 9973.7)	(8.2 – 7126)
	Median (IQR 25-75)	1186.2	1047.8	1368.0	1060.4	371.8
		(397.7 – 2616.5)	(580.7 – 2353.7)	(346.9 – 2660.3)	(267.4 – 6894.9)	(85.5 – 2036.6)
DNA integrity index	*p*-value	**<0.001**	**<0.001**	**0.018**	<**0.001**	
	Mean, Range	1.2	1.2	1.09	1.25	0.8
		(0.4 – 3.3)	(0.6 – 1.9)	(0.4 – 3.3)	(0.6 – 1.8)	(0.0 – 1.5)
	Median (IQR 25-75)	1.1	1.2	0.9	1.3	0.8
		(0.8 – 1.5)	(0.9 – 1.6)	(0.7 – 1.2)	(0.9 – 1.6)	(0.7 – 0.9)
Mesothelin, nM	*p*-value	0.242	**<0.001**	0.533	0.132	
	Mean, Range	36.9	104.2	8.0	5.5	7.4
		(1-392)	(4 – 392)	(1-31)	(0.7 – 24.5)	(1- 36)
	Median (IQR 25-75)	6.06	38.0	4.6	3.1	4.6
		(3.1 – 19.0)	(10.8 – 106.5)	(3 – 11.7)	(1.8 – 8.4)	(2.6 – 8.4)

*p-value-Mann-Whitney *U* test comparing diagnoses with benign effusions.

In cytology negative subjects eventually diagnosed with MPE (n=23), the DNA integrity index was significantly higher compared with that of subjects with benign effusions (median, 1.08 vs. 0.8, *p*<0.001).

Median pleural fluid DNA integrity index was not significantly different according to gender, smoking, pleural fluid biochemical or cytology classification in these subjects (Table 4).

**Table 4:** Comparison of subjects with malignant and benign pleural effusions

	**Pleural fluid DNA integrity index Median (IQR 25 - 75)**	***p*-value**
Diagnosis		**<0.001**
Malignant pleural effusion (n = 52)	1.1 (0.8 – 1.5)	
Non-malignant pleural effusion (n = 23)	0.8 (0.7 – 0.9)	
Gender		0.494
Female (n = 27)	0.95 (0.77 – 1.35)	
Male (n = 48)	0.94 (0.74 – 1.26)	
Smoking		0.996
Never (n = 25)	0.94 (0.79 – 1.28)	
Current/former smoker (n = 50)	0.95 (0.73 – 1.37)	
Light’s criteria classification		0.914
Transudate (n = 7)	0.91 (0.83 – 1.45)	
Exudate (n = 68)	0.96 (0.73 – 1.29)	
Pleural fluid cytology		0.082
Negative/Equivocal	0.90	
(n = 46)	(0.75 – 1.12)	
Positive	1.12	
(n = 29)	(0.75 – 1.56)	

*p-value-Mann-Whitney *U* test comparing diagnoses with benign effusions.

#### Serum

**Figure 4:**
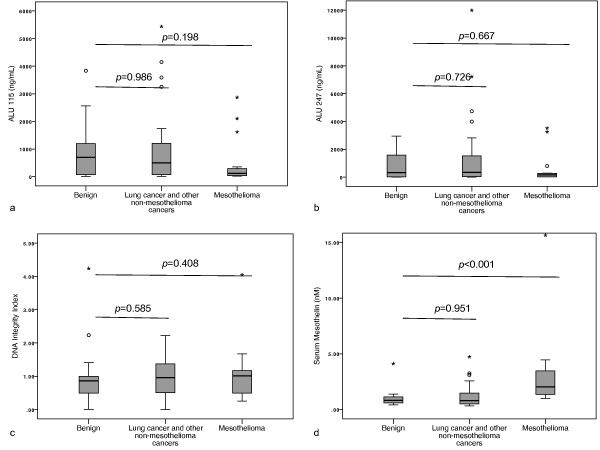
**Box plots of serum DNA fragments, integrity index and mesothelin concentrations in subjects with effusions due to benign aetiology, other cancers and mesothelioma.** DNA fragment values were determined by quantitative PCR for ALU (**a**) 115bp fragment and (**b**) a 247bp fragment. The DNA integrity index (**c**) was defined as the ratio of ALU 247/ALU 115 fragment levels. Mesothelin values (**d**) were determined by ELISA. Mann-Whitney *U* test to was used to assess for any differences in comparison to benign effusions and *p* values <0.05 were deemed significant.

Serum DNA fragment analysis results are provided in Table 5. There was no difference in serum ALU 115 (median, 234.4 ng/mL vs. 695.4 ng/mL, *p*=0.599) (Figure 4a), ALU 247 (median, 226.1 ng/mL vs. 330.2 ng/mL, *p*=0.968) (Figure 4b) or DNA integrity index (median, 0.9 vs. 0.9, *p*=0.461) (Figure 4c) between MPE and benign effusions.

**Table 5:** Serum DNA fragments, integrity index and mesothelin in subjects with malignant and benign pleural effusions

		**All MPE(*n*= 52)**	**MPM (n = 16)**	**Lung cancer (n=22)**	**Other cancers (n=14)**	**BPE(*n*= 23)**
ALU 115 (ng/ml)	*p*-value	0.599	0.198	0.855	0.777	
	Mean, Range	796.7 (6 – 5438)	503.7 (23 – 2869)	851.6 (6.3 - 4150.4)	1072.9 (31.8 – 5438.4)	826.8 (9 – 3835)
	Median (IQR 25-75)	234.4 (47.6 – 1107.9)	123.1 (36.5 – 321.4)	431.6 (47.1 – 1240.3)	500.2 (88.6 – 1113.8)	695.4 (59.1 – 1238.9)
ALU 247 (ng/ml)	*p*-value	0.968	0.667	0.955	0.512	
	Mean, Range	1088.3 (0 – 11995)	566.9 (10 – 3520)	1084.9 (0.1 – 7220.6)	1689.0 (11.7 – 11995.4)	930.7(2 – 2946)
	Median (IQR 25-75)	226.1 (31.6 – 1161.5)	157.7 (16.6 – 274.7)	333.6 (26.3 – 1458.4)	309.8 (50.7 – 1853.7)	330.2 (8.8 – 1832.7)
DNA integrity index	*p*-value	0.461	0.408	0.849	0.409	
	Mean, Range	1.0 (0 – 4.1)	1.1 (0.3 – 4.1)	0.9 (0.0 – 2.1)	1.1 (0.3 – 2.2)	1.0 (0 – 4.2)
	Median (IQR 25-75)	0.9 (0.5 – 1.3)	1.0 (0.5 – 1.2)	1.0 (0.4 – 1.3)	1.0 (0.6 – 1.5)	0.9 (0.5 – 1.0)
Mesothelin, nM	*p*-value	0.071	**<0.001**	0.692	0.552	
	Mean, Range	2.5 (0 - 32)	4.9 (1- 32)	1.2 (0 – 5)	1.2 (0.4 – 3.2)	1.0 (0 – 4)
	Median (IQR 25-75)	1.2 (0.7 – 2.2)	2.0 (1.3 – 3.5)	0.7 (0.5 – 1.3)	1.0 (0.6 – 1.6)	0.8 (0.5 – 1.2)

*p-value-Mann-Whitney *U* test comparing diagnoses with benign effusions.

There was no correlation between pleural fluid and serum DNA fragments or integrity index [Spearman’s rho, ALU 115, -0.023 (*p*=0.852), ALU 247, -0.009 (*p*=0.943); integrity index, -0.019 (*p*=0.876)].

### Mesothelin levels (pleural and serum)

**Figure 5:**
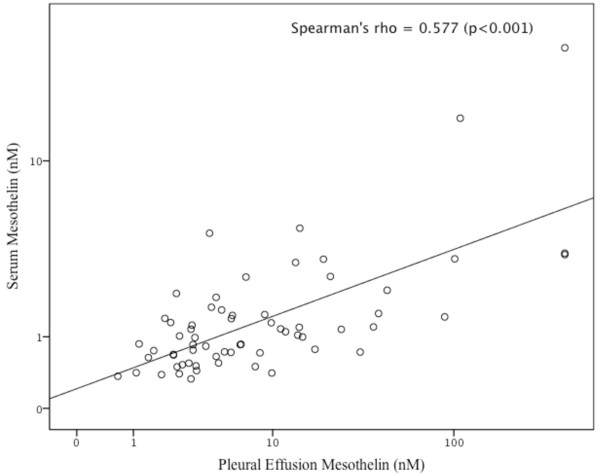
Dot plot representing the correlation of mesothelin (log values) concentration of pleural effusion and serum in study subjects (n=74).

Mesothelin was measured on 75 pleural fluid and 65 serum samples. There was insufficient serum for mesothelin testing in 10 subjects. Subjects with mesothelioma, in comparison to subjects with benign effusions, had significantly higher pleural fluid mesothelin (median 38 nM vs. 4.6 nM, *p*<0.001) (Figure 3d) and serum mesothelin levels (median, 2.0 nM vs. 1.0 nM, *p*<0.001) (Figure 4d). In the subjects with mesothelioma, there was a significant correlation between the serum and pleural fluid mesothelin levels (Spearman’s rho = 0.632, *p*<0.001) (Figure 5).

### Diagnostic utility of DNA integrity index and mesothelin

**Figure 6:**
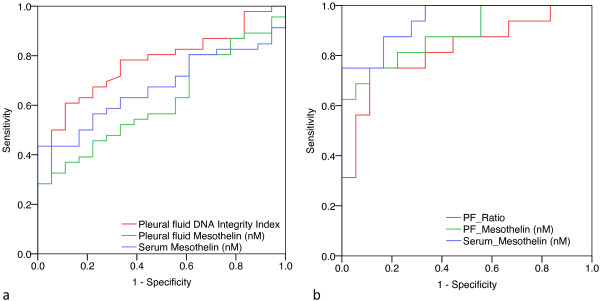
Receiver operator characteristic analysis of pleural effusion DNA integrity index, pleural effusion mesothelin and serum mesothelin to evaluate the diagnostic accuracy of malignant pleural effusions (a) and mesothelioma (b).

To determine the diagnostic utility of pleural effusion DNA integrity index we calculated its sensitivity and specificity using ROC analysis (Table 6). The area under the ROC curve for pleural effusion DNA integrity index was 0.766 (95% CI 0.646 – 0.866) (Figure 6a). We used a specificity threshold of 90% to compare the utility of pleural fluid DNA integrity index, pleural fluid mesothelin, and serum mesothelin in differentiating MPE from benign effusions. Pleural fluid DNA integrity index (cut-off level of 1.02) pleural fluid mesothelin (cut-off: 12.72 nM) and serum mesothelin (cut-off: 1.32 nM) distinguished MPE from benign effusions with sensitivity of 57%, 37% and 44% respectively. If cytology and raised pleural fluid DNA integrity index (>1.02) were considered together, the two tests showed complementarity increasing the sensitivity to 81% while maintaining a specificity of 87% in discriminating MPE from benign effusions. All of the three pleural effusion samples with false positive DNA integrity index were due to inflammatory pleuritis.

**Table 6:** Diagnostic accuracy of cytology, DNA integrity index and mesothelin

	**All malignant pleural effusions**	**Sensitivity (%)**	**Sensitivity (%)**	**Malignant pleural mesothelioma only**	**Sensitivity (%)**	**Sensitivity (%)**
	**Area under curve (95% CI)**			**Area under curve (95% CI)**		
Cytology		51	100		31	100
DNA Integrity index cut-off: 1.02	0.766 (0.646 - 0.866)	57	90	0.823 (0.676-0.970)	75	90
Pleural fluid mesothelin cut-off: 12.72 nM	0.620 (0.480-0.759)	37	90	0.885 (0.774-0.997)	75	90
Serum mesothelin Cut-off: 1.34 nM	0.674 (0.546-0.802)	44	90	0.941 (0.869-1.00)	75	90

We also evaluated the ability of pleural fluid DNA integrity index and mesothelin to distinguish mesothelioma from benign effusions (Figure 6b). Here we found that serum mesothelin provided the highest area under the ROC curve (0.94, 95% CI 0.87-1.00) followed by pleural fluid mesothelin (0.89, 95% 0.77-1.00) and pleural effusion DNA integrity index (0.82, 95% CI 0.68-0.97). Similarly, at a specificity of 90%, pleural fluid DNA integrity index (cut-off level of 1.06) pleural fluid mesothelin (cut-off: 12.91 nM) and serum mesothelin (cut-off: 1.34 nM) distinguished MPE from benign effusions with a sensitivity of 75% each respectively. When cytology, raised DNA integrity index and raised mesothelin were considered, the sensitivity increased from 31% for cytology alone to 81% (cytology plus DNA integrity index), 94% (cytology plus pleural fluid mesothelin) and 100% (cytology plus serum mesothelin). However the specificity decreased from 100% (cytology alone) to 70% for cytology plus pleural fluid mesothelin and 74% for cytology plus serum mesothelin, while it remained 87% for cytology plus DNA integrity index.

In subjects with cytology negative pleural effusions (n=46), MPE was eventually diagnosed in 23 (11 with mesothelioma, 8 with lung cancer, 2 with lymphoma and 2 with other cancers) (i.e. positive pleural tissue biopsy or disseminated malignancy with no alternative explanation for the effusion.). Serum mesothelin was available in 37 cytology negative subjects. Pleural fluid DNA integrity index was increased in 13 subjects (sensitivity 57%, specificity 87%, 81% PPV and 67% NPV), pleural fluid mesothelin was increased in 11 subjects (sensitivity 61%, specificity 84%, 78% PPV and 70% NPV) and serum mesothelin was raised in 11 subjects (sensitivity 48%, specificity 83%, PPV 73% and 61% NPV).

## Discussion

In this translational study, we evaluated the diagnostic utility of cfDNA fragments, DNA integrity index and mesothelin in the 75 matched pleural effusion and serum samples and found that pleural fluid DNA integrity index is higher in MPE (mesothelioma and other malignancies) compared to benign effusions. Importantly DNA integrity index had diagnostic utility in detecting MPE even in cytology negative effusions. In contrast, the diagnostic utility of pleural effusion and serum mesothelin was limited to mesothelioma but not non-mesothelioma MPEs.

It has been widely reported that total cfDNA in the plasma and serum is higher in cancer patients compared to healthy controls and may serve as a potential diagnostic biomarker for solid organ malignancies [9,15-20]. However the diagnostic utility of total cfDNA in plasma and serum for lung cancer has been questioned since the total cfDNA in of patients with non-malignant lung disease is not significantly different from that of lung cancer patients [18,21,22]. Additionally there is considerable variability in the reported values of total cfDNA in cancer patients, patients with non-malignant illnesses and healthy controls, limiting utility as a diagnostic biomarker [9]. Previous reports have demonstrated that cfDNA is 4-6 fold higher in serum compared to plasma [23] and maybe a better biological specimen to screen for cfDNA in malignancy [13]. Hence we used matched serum as a reference comparator to pleural fluid in evaluating the utility of cfDNA and DNA integrity index in diagnosing malignancy. An alternative approach to distinguish malignant from benign effusions has been to evaluate the utility of quantitative and qualitative tumour-specific alterations, such as microsatellite alterations in effusion DNA [24-28]. Economidou et al. studied patients with malignant (n=26) and benign (n=22) effusions and found that microsatellite instability and loss of heterozygosity in DNA from pleural fluid and blood were not diagnostically useful [27]. Conversely, Benlloch et al [24] and Chan et al [25] demonstrated that pleural effusion total DNA was significantly higher in MPE compared to benign effusions. However, in the study by Benlloch et al, all parapneumonic effusions were excluded from analysis and MPEs were compared predominantly to transudate effusions [24]. Chan et al. did not exclude parapneumonic effusions and found that MPEs had higher total DNA compared to both benign transudate effusions and infective effusions [25]. However they also found that while MPE total DNA in pleural fluid is significantly higher compared to transudate effusions (p<0.0001), it was less robustly significant compared to infective effusions (p=0.048) [25]. Additionally, in the group of infective effusions, the majority (82%) was due to tuberculosis, a subgroup that is not represented in our study.

Recently several studies have showed that in comparison to healthy controls, serum DNA integrity index is higher in breast tumours [29] and prostate tumours [30]. and plasma of head and neck tumours [31], naso-pharyngeal carcinoma [32] and rectal cancer [33]. In contrast we did not find a difference in serum DNA integrity index between subjects with MPE and benign effusions. This is most likely because of our subjects with inflammatory pleuritis and parapneumonic effusions accounted for 35% and 17% of the benign effusion cohort. Our findings are consistent with the findings of Schmidt et al, who also did not find a difference in the serum DNA integrity index of lung cancer patients compared to those with non-malignant lung disease [34]. This is most likely due to the release of increased quantities of DNA into the blood from both apoptotic and necrotic cells in severe inflammatory processes [35].

To our knowledge, only one other study by Salani et al, has evaluated the diagnostic utility of DNA integrity index in effusions [26]. They measured the integrity index of cyclin E, a gene frequently amplified in ovarian carcinoma in ascites and pleural effusions. This study demonstrated that cyclin E assay was specific to ovarian carcinomas, thereby limiting its usefulness as a diagnostic test for all MPEs. In our study we used ALU repeats to measure pleural effusion DNA integrity index, since ALU repeats are ubiquitous and the ALU qPCR assay has previously been validated in several different malignancies [13,29,33,36].

Cytology remains the diagnostic standard for evaluating pleural effusion samples. However it is can be difficult to establish a diagnosis of MPE in subjects with cytology-negative effusions, since in such circumstances diagnosis requires invasive pleural biopsy tests, such as thoracoscopy. We found that when combined with cytology, pleural fluid DNA integrity index substantially increased the ability to distinguish benign effusions from MPE (sensitivity 81% vs. 55%) and mesothelioma (sensitivity. 81% vs. 31%). Importantly, almost a third of false positive pleural fluid DNA integrity index results were due to parapneumonic effusions. Additionally, we showed that in cytology negative effusions, elevated pleural fluid DNA integrity index had 81% PPV. Hence pleural fluid DNA integrity index provides valuable additional information to pleural fluid cytology, particularly in subjects with either inconclusive or “suspicious” cytology results. This may have clinical implications since an elevated pleural fluid DNA integrity index in a cytology-negative subjects should be prioritized to undergo thoracoscopy.

Our study included subjects with mesothelioma (31% of subjects with MPE), a subgroup that was present in only 0-3% of other studies that have studied total DNA in effusions [24-26]. It is now important to identify accurate diagnostic biomarkers for mesothelioma since it is increasing in incidence, with a peak predicted to occur between 2014 to 2021 [37]. Consistent with previous studies, we found that pleural effusion and serum mesothelin has diagnostic utility for mesothelioma [7,8,38]. However a weakness of our findings is that serum mesothelin results could not be obtained in 13% (10/75) of study subjects. Nonetheless, our data demonstrate that pleural fluid DNA integrity index is comparable in sensitivity to pleural fluid and serum mesothelin in diagnosing mesothelioma.

There are certain limitations to consider before the results of our study can be applied to other patient populations. Firstly, while our study has found that DNA integrity has utility in distinguishing MPEs from benign effusions, it should be noted the method used for measuring DNA integrity in our study is one of several others that have been reported in the literature [12]. In this study, we have used the ratio of the longer DNA fragments to the shorter DNA fragments to measure DNA integrity (ALU 247/115), as described by Umetani et al [13]. This methodology has since been used by other investigators to measure DNA integrity in the serum of breast cancer patients [36] and plasma of rectal cancer patients [33]. Most recently, Agostini et al found that DNA integrity (ALU 247/115) in the plasma of patients with rectal adenocarcinoma (n=67, median, IQR25-75 1.1 (0.7-1.9)) compared to healthy control subjects (n=35, median, IQR25-75; 0.1 (0.0 – 0.4)) [33]. The findings of DNA integrity in malignancy are similar to our study where the median DNA integrity in serum 0.9 (0.5-1.3). Interestingly, the serum DNA integrity index in our cohort of subjects with benign effusions was also relatively high and not significantly different from subjects with MPEs (median, IQR25-75; 0.9, 0.5-1.0). This is not unexpected since our ‘control’ population were not normal healthy volunteers but subjects with non-malignant pulmonary diseases. Secondly the different studies have not only used other methods to measure DNA integrity, they have also used other protocols to process and extract DNA from blood [12]. Recently Fleischhacker et al found that significantly different amounts of absolute DNA values were obtained from plasma using different DNA isolation methods [39]. Indeed Jung et al, note that the considerable heterogeneity in preanalytical and analytical factors considerably determine the interpretation of cfDNA and DNA integrity studies in malignancy, thereby limiting the translation of this test into clinical practice [12]. Hence, despite extensive research into the utility of cfDNA and DNA integrity index as diagnostic and prognostic biomarkers in malignancy, the methodological discrepancies have dictated that the tests have remained as research tools. Thirdly, another limitation of our study relates to the relatively small number of subjects evaluated and the casemix of pleural effusion diagnoses, particularly the relatively high proportion of mesothelioma subjects (31%), derived at a single Australian thoracic tertiary referral centre. It is well recognised that the distribution of aetiologies of pleural effusions (including MPEs) vary considerably according to geographic location [40]. Further validation will be needed to address issues of generalisability and applicability. Finally, another restriction of our study is that the DNA extracted from a mixture of various types of cells present in pleural fluid (tumour cells, mesothelial cells, white blood cells). The influence of non-tumour cells on DNA fragments is currently not known and will also need to be evaluated. For these reasons, our findings should be interpreted as a pilot study, albeit with results suggesting that pleural fluid DNA integrity index is a promising diagnostic biomarker for MPEs. The results require confirmation in prospectively conducted multicentre studies with sufficient attention paid to the methodological variations common in cfDNA and DNA integrity index research before they can be incorporated into clinical decision-making algorithms.

## Conclusions

Our results indicate that pleural fluid DNA integrity index is a promising diagnostic biomarker for MPEs. The increased sensitivity over conventional cytological examination may assist clinicians in directing patients for further examinations, such as thoracoscopy or to adopt a conservative approach. Pleural fluid DNA integrity index maybe a valuable adjunctive test when evaluating subjects presenting with pleural effusions, especially when cytology is inconclusive or suspicious. These findings warrant confirmation and consequently, we are planning a large-scale prospective study to evaluate the clinical utility of pleural fluid DNA integrity index and mesothelin as initial tests in patients with pleural effusions. The introduction of pleural fluid DNA integrity index, a minimally invasive and technically simple test can provide additional information that may reduce the need for thoracoscopy, an invasive test with associated patient-related morbidity.

## Abbreviations

DNA: Deoxyribonucleic acid; DNA-cell-free: Deoxyribonucleic acid; MPE: Malignant pleural effusions; qPCR: Quantitative polymerase chain reaction; ELISA: Enzyme-linked immunosorbent assay (ELISA).

## Competing interests

None of the authors have any competing interests to declare.

## Authors’ contributions

Conceived and designed experiments: KBS, VR, RVB, IAY, KMF. Performed the experiments: KBS. Analysed the data: KBS, VR, RVB, IAY, KMF. Wrote the manuscript: KBS. Sample collection: MNW, KSM, RN. Cytology and pathology analysis: BEC, EED. Obtained informed consent from patients: LP, EM, DC. All authors read and approved the final manuscript.

## Pre-publication history

The pre-publication history for this paper can be accessed here:

http://www.biomedcentral.com/1471-2407/12/428/prepub
